# Unveiling the interplay between microbiota and PD1/PD-L1 axis in tumor immunity and immunotherapy

**DOI:** 10.3389/fimmu.2025.1690374

**Published:** 2025-11-27

**Authors:** Qiguang Lu, Jiasheng Wu, Xiaoyan Yu, Juanjuan Qian, Zhengwei Song

**Affiliations:** 1 Department of Respiratory Medicine, The Second Affiliated Hospital of Jiaxing University, Jiaxing, Zhejiang, China; 2 Department of Surgery, The Second Affiliated Hospital of Jiaxing University, Jiaxing, Zhejiang, China

**Keywords:** microbiota, PD1/PD-L1 axis, tumor immunity, cancer immunotherapy, tumor microenvironment

## Abstract

Microbial communities across diverse body sites critically shape host immunity and tumor responses. Within this framework, the PD-1/PD-L1 axis emerges as a central pathway governing tumor immune evasion and resistance to therapy. Recent evidence reveals that microbiota—from the gut, lungs, and elsewhere—significantly influence PD-1/PD-L1 signaling, thereby altering immune checkpoint blockade efficacy. This review synthesizes current understanding of the microbiota-PD-1/PD-L1 interplay, examining how microbial composition and metabolites impact immune cell activity, the tumor microenvironment, and immunotherapy outcomes. We detail mechanisms through which microbiota regulate PD-1/PD-L1 expression, fostering immune tolerance and tumor progression while modulating therapeutic responses. The translational potential of microbiota-targeted strategies to enhance PD-1/PD-L1 therapy and overcome resistance is discussed. Integrating microbiota modulation with existing immunotherapies offers promising avenues for precision cancer treatment. Advancing these concepts into clinical practice will require future research to establish microbiome-based interventions as transformative tools in oncology.

## 1 Introduction

The human microbiota, composed of trillions of microorganisms residing in various body sites, plays an essential role in maintaining homeostasis and influencing a wide range of physiological processes, including immune system function (1–4). Among the diverse microbial communities, the gut microbiota has received the most attention due to its profound impact on immune modulation, yet emerging evidence suggests that microbiota residing in other body niches, such as the skin, lungs, and even the tumor microenvironment (TME), also play crucial roles in immune regulation (5–8). The microbiota can influence immune responses through direct interactions with immune cells, as well as by producing metabolites that impact systemic inflammation and immune cell function (9–11).

In recent years, it has become increasingly clear that the microbiota plays a significant role in cancer immunity, especially in modulating the efficacy of immunotherapies. One of the most critical immune pathways involved in tumor immune evasion is the programmed cell death protein 1 (PD1) and its ligand (PD-L1) axis (12, 13). PD1/PD-L1 is a key immune checkpoint that suppresses T cell activation and promotes immune tolerance, allowing tumors to evade immune surveillance (14, 15). The blockade of the PD1/PD-L1 pathway has revolutionized cancer immunotherapy, but its effectiveness varies widely among patients, with some experiencing durable responses while others develop resistance (16–18).

Recent studies have suggested that the microbiota may influence the PD1/PD-L1 axis, thereby affecting the immune response within the TME (19–21). Microbial modulation of immune cell activation, tumor progression, and therapeutic outcomes could potentially explain some of the variability in the response to immune checkpoint inhibitors (ICIs) (22–28). While much of the research has focused on gut microbiota, increasing evidence points to a broader impact of microbiota from various body sites in shaping tumor immunity and modulating immune checkpoint pathways (29–31). Understanding how microbiota impact PD1/PD-L1 signaling and immune checkpoint blockade may open new avenues for enhancing cancer immunotherapy.

This review aims to provide a comprehensive overview of the complex interplay between microbiota and the PD1/PD-L1 axis, exploring the mechanisms through which microbiota influence immune cell function, tumor progression, and the response to immunotherapy. We will discuss the emerging role of microbiota as modulators of PD1/PD-L1 expression and their potential in overcoming resistance to ICIs. By investigating the dynamic relationship between microbiota and tumor immunity, we propose new strategies for precision cancer immunotherapy that integrate microbiome modulation with conventional immune checkpoint blockade therapies.

## 2 Microbiota and tumor immunity

**Figure 1:**
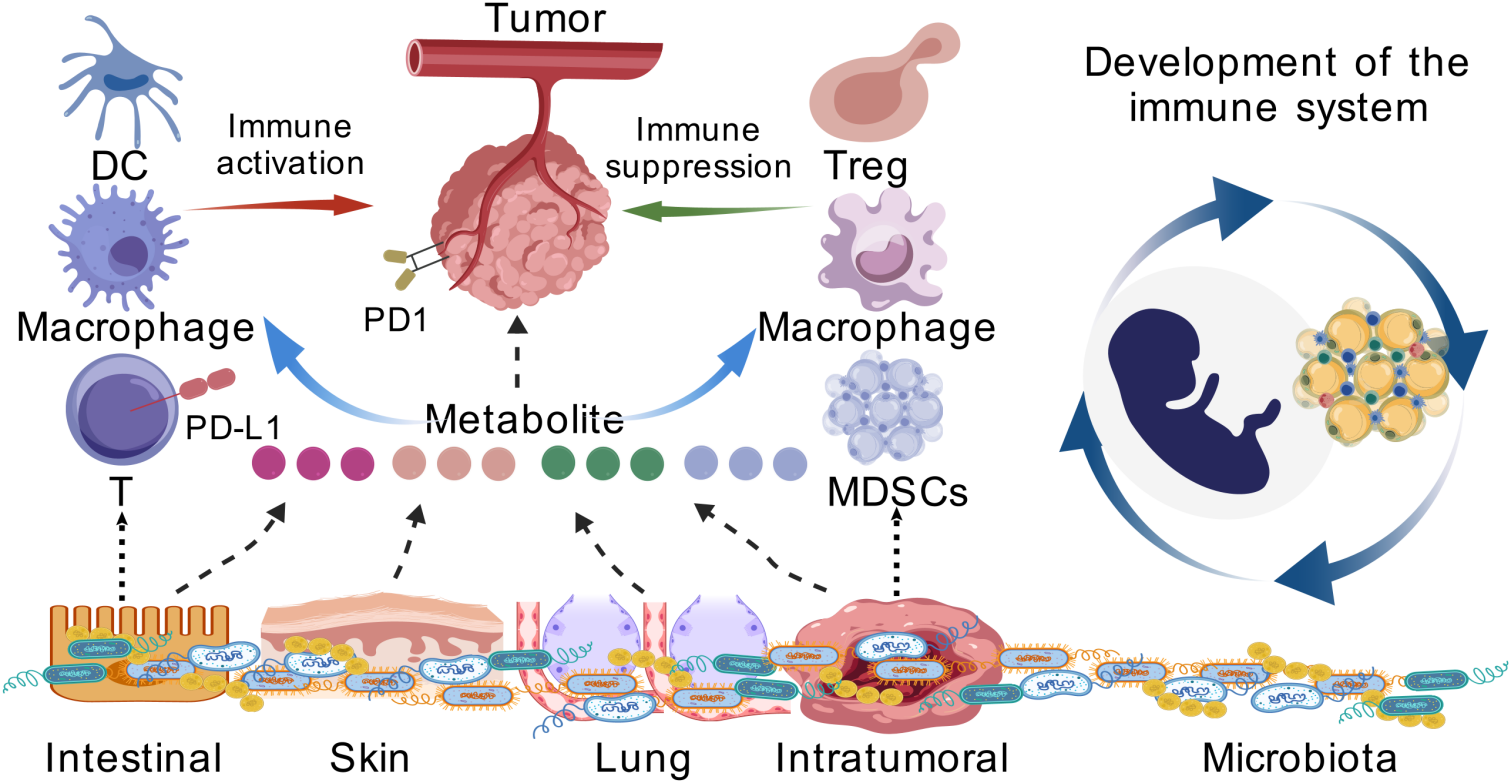
Microbiota and tumor immunity. The development and function of the immune system are closely influenced by microbial colonization, especially in the early stages of life. Against the backdrop of cancer, the gut microbiota regulates the immune composition of the TME. Changes in the immune cell population induced by microorganisms and their metabolites can affect the immune system’s ability to recognize and eliminate tumor cells. Microorganisms from the intestines, skin, lungs and even the tumors themselves have been proven to affect tumor immunity by regulating the PD1/PD-L1-related pathways.

The relationship between microbiota and tumor immunity has become an exciting area of research in recent years. The microbiota, which consists of trillions of microorganisms residing in various body sites, plays a crucial role in regulating the host’s immune system. Growing evidence has highlighted the significant impact of microbial communities, especially the gut microbiota, in shaping immune responses and influencing the development and progression of tumors (**Figure 1**).

### 2.1 Immune modulation by microbiota

Microbiota, through both direct and indirect interactions, plays an essential role in modulating the host’s immune system. The immune system’s development and function are closely influenced by microbial colonization, especially early in life (32–34). The gut microbiota is particularly important in shaping the immune system’s balance, including the distribution and function of immune cells such as regulatory T cells (Tregs), dendritic cells, and macrophages (35–40). These immune cells are key players in maintaining immune homeostasis, tolerance, and inflammation control (41, 42).

Microbiota influences immune responses by producing metabolites that directly impact immune cells (43, 44). Short-chain fatty acids (SCFAs), such as butyrate, propionate, and acetate, are metabolites produced by the fermentation of dietary fibers by gut bacteria (45–48). SCFAs have been shown to have significant anti-inflammatory effects and can promote the differentiation of Tregs, which help maintain immune tolerance and prevent excessive inflammation (49–53). These metabolites also influence the activity of dendritic cells and T cells, promoting immune responses against pathogens and tumors (54, 55).

In the context of cancer, the gut microbiota regulates the immune composition of the TME (56, 57). Microbial-induced changes in immune cell populations can influence the efficacy of the immune system in recognizing and eliminating tumor cells (58, 59). For instance, the balance between effector T cells and Tregs, as well as the infiltration of antigen-presenting cells, plays a crucial role in tumor immunity. Dysbiosis, an imbalance in microbial communities, can impair immune responses, contributing to immune escape and tumor progression (60–63). As a bridge connecting microbial signals to adaptive immunity, the function of innate lymphoid cells (ILCs) exhibits remarkable plasticity, serving as a classic example of context-dependency. For instance, while ILC3s typically maintain intestinal barrier integrity by producing IL-22, under conditions of chronic inflammation or specific microbial stimulation (such as from certain pathogenic bacteria), they may convert to a pro-inflammatory phenotype, producing abundant IL-17 and thereby promoting the development and progression of colorectal cancer (64). Conversely, in non-small cell lung cancer, the presence of ILC3s is associated with the formation of tertiary lymphoid structures and a favorable prognosis (64). This indicates that ILC function is not fixed but is “reprogrammed” by the local tissue microenvironment and specific microbial signals. Consequently, the effect of microbes on the PD-1/PD-L1 axis via ILCs is highly dependent on the functional state of the ILCs within the specific tumor context.

### 2.2 Mechanisms of immune evasion in tumors

Tumors have evolved various mechanisms to escape detection and destruction by the host immune system, allowing for continuous growth and metastasis (65, 66). One of the most important mechanisms of immune evasion is the upregulation of immune checkpoint molecules, including PD1 and its ligand PD-L1 (67, 68). PD1/PD-L1 signaling inhibits T cell activation, thereby suppressing immune responses and promoting immune tolerance within the TME (69–71). This immune checkpoint pathway is exploited by tumors to evade immune surveillance (72, 73).

PD1/PD-L1 is an important immune checkpoint pathway. PD1 is mainly expressed on the surface of T cells and other immune cells, while its ligand PD-L1 is expressed on the surface of tumor cells and immune cells (74–76). By binding PD1 to PD-L1, T cell activation is inhibited, leading to immune tolerance and promoting tumor cells to escape immune surveillance (77, 78). This pathway plays a key role in tumor immune escape, inhibiting T cell function and weakening anti-tumor immune response. ICIs, such as anti-PD1 and anti-PD-L1 antibodies, have achieved significant efficacy in a variety of cancer treatments, but still face limitations such as mixed efficacy and immune-related side effects, which has driven deeper research to optimize their application and overcome resistance (79–81).

In addition to immune checkpoints, tumors can alter the TME by influencing immune cell recruitment, cytokine production, and immune cell dysfunction (82–87). The TME is characterized by various immune cells, including macrophages, myeloid-derived suppressor cells (MDSCs), and Tregs, which support tumor growth and immune suppression (66, 88). These immune cells can further enhance the tumor’s ability to escape immune surveillance and resist immune therapies.

### 2.3 The role of microbiota in tumor immune evasion

Recent research has revealed that microbiota plays a significant role in tumor immune evasion by modulating immune checkpoint pathways, particularly the PD1/PD-L1 axis. The composition of the microbiota, especially gut microbes, can directly or indirectly affect immune responses, influencing how tumors evade immune surveillance. Specific microbial species can alter the expression of PD-L1 in tumor cells, immune cells, or both. For example, certain beneficial microbes such as Bifidobacterium have been found to modulate the immune system, leading to reduced tumor growth and enhanced immune responses (89–91).

In addition to modulating immune cells, microbiota-derived metabolites such as SCFAs can influence immune checkpoint regulation. Butyrate, for example, has been shown to enhance T cell function and promote the expression of PD-L1 in certain immune cell populations, thus playing a role in immune evasion within the TME (92–94). Furthermore, microbial metabolites can influence the production of pro-inflammatory cytokines and other signaling molecules that enhance or suppress immune responses, impacting the overall immune landscape within tumors (95, 96).

Interestingly, the interaction between microbiota and immune checkpoint signaling is not limited to the gut. Microbial species from other body sites, such as the skin, lungs, and even the tumor itself, have been shown to impact tumor immunity (97–101). For instance, the lung microbiome has been associated with the regulation of PD-L1 expression in lung cancer, while skin microbiota has been linked to immune responses in melanoma (102–104). This suggests that microbiota from various niches can collectively contribute to the modulation of immune checkpoints and tumor immune evasion.

## 3 Mechanisms of microbiota modulating PD1/PD-L1 axis​

**Figure 2:**
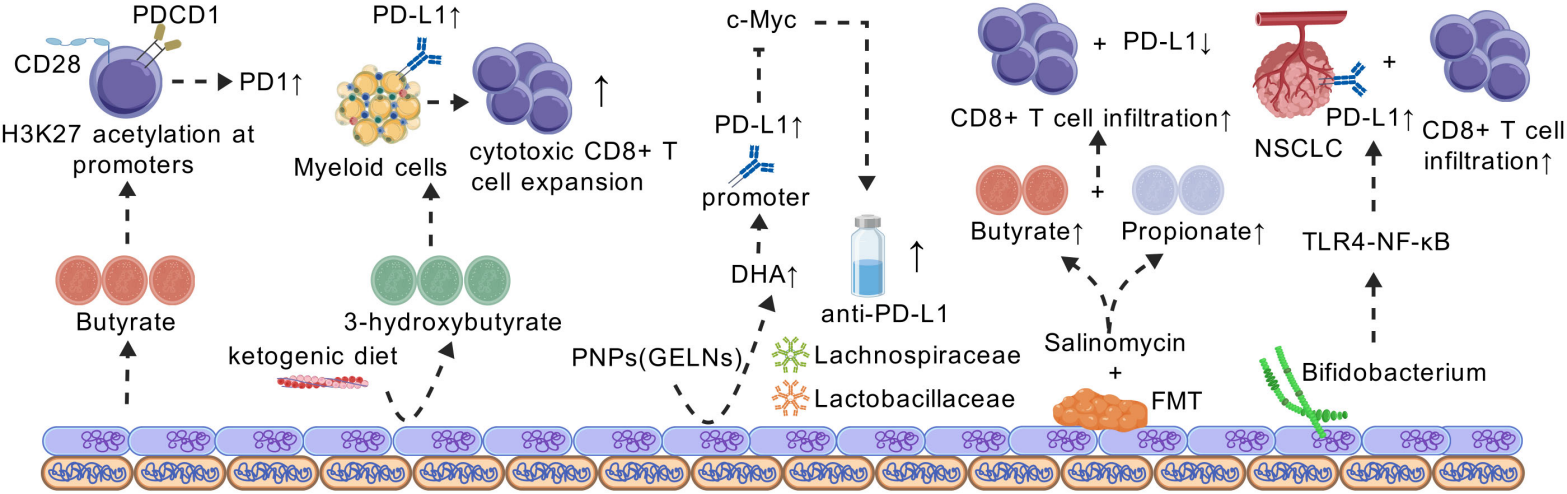
Mechanisms of microbiota modulating PD1/PD-L1 axis. Butyrate increases PD-1 expression and enhances the anti-PD-1 therapeutic response by enhancing histone H3K27 acetylation on the promoters of PDCD1 and CD28 in CD8+ T cells. The 3HB from KD inhibits the upregulation of PD-L1 in myeloid cells induced by immunotherapy and simultaneously promotes the expansion of CTLs. Plant-derived nanoparticles GELNs can enrich DHA through the regulation of intestinal bacteria (such as the Conidae and Lactobacillus families). DHA binds to the PD-L1 promoter and inhibits its transcription by antagonizing c-Myc, thereby making the tumor sensitive to PD-L1 inhibitors. FMT combined with salinomycin increases the levels of propionate and butyrate, promotes the infiltration of CD8+ T cells, and reduces the expression of PD-L1. The extracellular vesicles derived from Bifidobacterium upregulate PD-L1 through the TLR4-NF-κB pathway and simultaneously enhance the infiltration of CD8+ T cells.

The newly emerged evidence highlights the multi-faceted role of the gut microbiota in regulating the PD1/PD-L1 axis. These regulatory effects make the tumor sensitive or insensitive to PD1/PD-L1 blockade. Understanding these approaches provides crucial insights for overcoming immunotherapy resistance and optimizing clinical outcomes through microbial intervention (**Figure 2**).

### 3.1 Microbiota regulation of PD1/PD-L1 via the immune system

Emerging evidence highlights the pivotal role of gut microbiota in modulating immune cell activity to influence PD1/PD-L1 axis dynamics. For instance, specific gut microbial communities enhance the efficacy of anti-PD-1 therapy by downregulating the PD-L2-RGMb pathway, a newly identified immunosuppressive axis. Blocking PD-L2-RGMb interactions or deleting RGMb in T cells significantly restored anti-PD-1 responsiveness in previously resistant models, including germ-free mice and fecal microbiota-transplanted mice, indicating microbiota-driven remodeling of the tumor immune microenvironment (TIM) (105). Similarly, the ketogenic diet (KD) and its metabolite 3-hydroxybutyrate (3HB) suppress myeloid cell PD-L1 upregulation induced by immunotherapy while expanding CXCR3+ T cell populations, thereby enhancing anti-PD-1/CTLA-4 efficacy (106). Additionally, interventions like MS-20 (Symbiota^®^), a postbiotic, amplify CD8+ T cell infiltration into tumors and reduce PD1 expression, correlating with increased abundance of Ruminococcus bromii (107). Estrogen pretreatment in MC38 colon cancer models also shifts gut microbiota composition (e.g., reducing Enterobacteriaceae and enriching Lactobacillus), which may synergize with anti-PD-L1 therapy by altering immune cell dynamics (108). These findings collectively underscore microbiota’s capacity to orchestrate immune cell functions—spanning T cells, myeloid cells, and macrophages—to regulate PD1/PD-L1 signaling.

### 3.2 Microbial metabolites in PD1/PD-L1 pathway modulation

Gut microbiota-derived metabolites serve as critical mediators linking microbial activity to PD1/PD-L1 axis regulation. Butyrate, a SCFA, exemplifies this mechanism by enhancing histone H3K27 acetylation at the PDCD1 and CD28 promoters in CD8+ T cells, thereby elevating PD-1 expression and amplifying anti-PD-1 therapeutic responses in non-small cell lung cancer (NSCLC) (109). Similarly, 3HB from KD inhibits immunotherapy-induced PD-L1 upregulation in myeloid cells while promoting cytotoxic CD8+ T cell (CTL) expansion (106). Plant-derived nanoparticles (PNPs), such as ginger exosome-like nanoparticles (GELNs), enrich docosahexaenoic acid (DHA) via gut bacterial modulation (e.g., Lachnospiraceae and Lactobacillaceae). DHA binds to the PD-L1 promoter, suppressing its transcription by antagonizing c-Myc, thereby sensitizing tumors to PD-L1 inhibitors (110). Fecal microbiota transplantation (FMT) combined with salinomycin in colorectal cancer (CRC) models elevates propionate and butyrate levels, fostering CD8+ T cell infiltration and reducing PD-L1 expression (111). These metabolites act as molecular bridges, directly or indirectly fine-tuning immune checkpoint dynamics.

Nevertheless, interpreting the functions of microbial metabolites requires caution, as growing evidence indicates their effects are highly context-dependent and can even exhibit seemingly paradoxical dual roles. Taking the short-chain fatty acid butyrate as an example, this review mentions in several sections that it enhances CD8+ T cell function through epigenetic mechanisms, thereby improving responses to immunotherapy. Conversely, however, multiple studies have also shown that butyrate can induce an immunosuppressive state by promoting the differentiation and function of regulatory T cells (Tregs) or by directly upregulating PD-L1 expression on tumor cells and myeloid cells (51, 112, 113). This seemingly contradictory dual role is likely determined by the type of tumor and the local cytokine environment. In a microenvironment dominated by Th1-type cytokines (such as IFN-γ, IL-12), butyrate may be more inclined to enhance the function of effector T cells; while in a microenvironment enriched with TGF-β and other signals, it may drive immunosuppression. Similarly, bile acids can exert anti-inflammatory effects locally in the intestinal tract through the Farnesoid X receptor, but their elevated systemic levels are associated with immunosuppression in hepatocellular carcinoma (114). Such discrepancies may stem from various factors, including the local concentration of the metabolite, duration of exposure, the specific tumor microenvironment (e.g., cytokine milieu and immune cell composition), and the tumor type itself. Similarly, the role of specific bacterial species is not fixed. For instance, microbial communities that are beneficial in immunocompetent individuals may act as opportunistic pathogens in immunocompromised hosts, and their interactions with different anticancer regimens (such as chemotherapy and radiotherapy) may yield complex outcomes that cannot be predicted from immune checkpoint inhibitor models alone. These paradoxes highlight the complexity of the field and caution against simplistically categorizing microbes or their metabolites as purely “beneficial” or “detrimental.” Instead, there is a need to elucidate the precise conditions under which they exert specific functions.

### 3.3 Microbial modulation of PD1/PD-L1 signaling pathways

Microbiota and their components engage specific signaling cascades to regulate PD1/PD-L1 expression. The PD-L2-RGMb axis, suppressed by commensal bacteria, represents a novel pathway through which microbiota enhance anti-PD-1 efficacy by alleviating T cell exhaustion (105). Bifidobacterium-derived extracellular vesicles (Bif.bev) exploit the TLR4-NF-κB pathway to upregulate PD-L1 in NSCLC cells while paradoxically enhancing CD8+ T cell infiltration, suggesting context-dependent modulation of immune checkpoints (115). GELN-derived aly-miR159a-3p inhibits bacterial phospholipase C, leading to DHA accumulation and subsequent PD-L1 transcriptional repression via c-Myc blockade (110). Furthermore, butyrate activates TCR signaling in CD8+ T cells, augmenting cytotoxic cytokine production and PD-1/PD-L1 checkpoint engagement (109). These mechanisms highlight the intricate interplay between microbial signals, host epigenetic regulation, and immune checkpoint pathways, offering actionable targets for overcoming immunotherapy resistance. However, the immunomodulatory effects mediated by *Bif. bev* exhibit significant context-dependency. The paradoxical effect of “upregulating PD-L1 while simultaneously enhancing anti-tumor immunity” observed in NSCLC models may stem from the unique composition of the tumor immune microenvironment. For instance, in an immunosuppressive microenvironment rich in myeloid-derived suppressor cells or regulatory T cells, the upregulation of PD-L1 might dominate and impair the anti-tumor response. Conversely, in an inflammatory microenvironment with pre-existing, pre-activated T cells, *Bif. bev* may primarily function as a “brake,” preventing excessive immune activation, thereby synergizing with and enhancing the efficacy of checkpoint inhibitors. Furthermore, differences such as bacterial subtypes, the dosage of vesicles, and their routes of administration are also key factors contributing to the discrepant observations across different studies (116). Therefore, categorizing *Bifidobacterium*or its products simply as “beneficial” or “detrimental” is an oversimplification; their immunological and microbiological context must be taken into account.

## 4 Microbiota and PD1/PD-L1 immunotherapy ​

### 4.1 Gut microbiota composition as a determinant of immunotherapy efficacy

Clinical and preclinical studies have established a strong correlation between specific gut microbial taxa and enhanced responses to anti-PD1/PD-L1 therapy. For instance, responders to PD-1 blockade exhibit higher abundances of Parabacteroides distasonis and Bacteroides vulgatus, which are associated with elevated levels of the microbial metabolite valerate. Valerate reduces immunosuppressive metabolites like L-kynurenine and the kynurenine/tryptophan (Kyn/Trp) ratio, thereby suppressing Tregs and activating effector T cells (117). Similarly, FMT from responders to refractory melanoma patients reshapes gut microbial communities, enriching immunostimulatory taxa (e.g., Clostridiales and Ruminococcaceae) while reducing IL-8-expressing MDSCs. This remodeling enhances CD8+ T cell activation and TME immunogenicity, achieving clinical benefit in 40% of previously resistant patients (56). Akkermansia muciniphila and its outer membrane protein Amuc_1100 further exemplify beneficial microbiota, promoting CTL expansion in colitis-associated CRC by downregulating PD-1 and upregulating TNF-α in mesenteric lymph nodes (118). Conversely, Fusobacterium nucleatum and its metabolite succinate drive resistance in CRC by inhibiting the cGAS–IFN-β pathway, impairing CD8+ T cell recruitment. Depleting F. nucleatum via metronidazole restores PD-1 inhibitor sensitivity, highlighting its role as a predictive biomarker for resistance (119).

### 4.2 Microbial drivers of immune checkpoint inhibitor resistance

Resistance to PD1/PD-L1 therapy is closely linked to dysbiotic gut microbiota and their metabolites. In CRC, Fusobacterium nucleatum-derived succinate suppresses CD8+ T cell infiltration by inhibiting cGAS–STING signaling, establishing an immunosuppressive TME (119). Similarly, gut microbiota alterations in non-responders to PD-1/PD-L1 inhibitors are characterized by reduced Faecalibacterium prausnitzii abundance. Low F. prausnitzii levels exacerbate ICIs-induced colitis and weaken anti-tumor immunity, whereas its supplementation alleviates intestinal inflammation while enhancing CTL activity and tumor control (120). In biliary tract cancer, poor clinical outcomes correlate with Bacilli, Lactobacillales, and Pyrrolidine, which are enriched in non-durable clinical benefit patients. These taxa are associated with suppressed anti-tumor immunity and shorter survival, contrasting with the survival-promoting effects of Alistipes in responders (121). Moreover, dysregulated microbial metabolism contributes to resistance: low butyrate-producing microbiota in non-responders reduce histone H3K27 acetylation in CD8+ T cells, dampening PD-1 expression and T cell receptor (TCR) signaling (122).

### 4.3 Modulating gut microbiota to overcome resistance and enhance efficacy

Targeted modulation of gut microbiota emerges as a promising strategy to improve PD1/PD-L1 immunotherapy outcomes. Probiotics and postbiotics: Lactobacillus gallinarum produces indole-3-carboxylic acid (ICA), which inhibits IDO1-mediated kynurenine production and blocks aryl hydrocarbon receptor (AHR)-dependent Treg differentiation, thereby restoring CD8+ T cell cytotoxicity and sensitizing CRC to PD-1 inhibitors (123). Dietary interventions: Inulin gel enriches SCFA-producing commensals (e.g., Bifidobacterium), fostering stem-like TCF1+PD-1+CD8+ T cells in the TME and enhancing systemic IFN-γ+CD8+ T cell responses (124). Similarly, KD or supplementation with 3HB suppress PD-L1 upregulation in myeloid cells while expanding CXCR3+ T cells, synergizing with anti-PD-1 therapy (125). Pharmacological and microbial engineering: CBM588, a microbial modulator, increases Ruminococcaceae abundance and IL-10 production, improving gut immune homeostasis and overcoming PD-1 blockade resistance in murine models (126). FMT: Transferring responder microbiota to refractory patients or germ-free mice restores anti-PD-1 responsiveness by elevating beneficial taxa (e.g., Akkermansia) and SCFAs like propionate and butyrate, which promote CD8+ T cell infiltration and suppress PD-L1 (56, 127).

### 4.4 Non-gut microbiota and their impact on PD-1/PD-L1 regulation

While the gut microbiota has been extensively studied, emerging evidence underscores the critical role of non-gut microbiota—including intratumoral, lung, skin, and oral microbes—in shaping antitumor immunity and modulating the PD-1/PD-L1 axis. These microbial communities reside directly within tumor tissues or at barrier sites, allowing them to interact with local immune cells and influence checkpoint pathways through context-specific mechanisms. For instance, in lung cancer, intratumoral fungi such as *Aspergillus sydowii* recruit myeloid-derived suppressor cells (MDSCs) and induce an immunosuppressive microenvironment, thereby upregulating PD-L1 expression on tumor cells and promoting resistance to anti-PD-1 therapy (128). Similarly, intratumoral bacteria in lung adenocarcinoma—including butyrate-producing species like *Roseburia*—can enhance metastasis by stimulating PD-L1 via metabolite-driven pathways (e.g., butyrate-induced H19/MMP15 signaling) and polarizing macrophages toward an M2 phenotype (129). Beyond the lungs, intratumoral microbes in gastric cancer, such as *Fusobacterium nucleatum*, recruit tumor-associated neutrophils (TANs) and activate IL-17/NF-κB pathways to foster PD-L1-dependent immune evasion (130). Even in melanoma, skin microbiota and intratumoral fungi have been linked to differential T-cell infiltration and PD-1 expression, though their mechanisms are less defined (131, 132). Importantly, these non-gut microbes often operate independently of gut-centric effects, as demonstrated by single-cell analyses showing direct microbial-host interactions in tumor tissues that alter gene expression in T cells and macrophages. The therapeutic implications are significant: for example, targeting intratumoral fungi with antifungals or engineering microbes to deliver PD-1/PD-L1 modulators could overcome local resistance. However, challenges remain, including the low biomass of non-gut microbiota and the need for spatially resolved techniques to dissect their niche-specific functions. Future studies should prioritize mapping microbial influences across all body sites to develop holistic interventions that integrate gut and non-gut microbiota for precision immunotherapy (132).

### 4.5 Microbial metabolites as predictive biomarkers and therapeutic targets

Microbiota-derived metabolites serve as both biomarkers and mediators of immunotherapy efficacy. High serum butyrate levels correlate with improved PD-1 inhibitor responses in NSCLC by enhancing PDCD1 and CD28 promoter acetylation in CD8+ T cells (122). Conversely, succinate accumulation from F. nucleatum predicts resistance in CRC, while methylglyoxal (MG), a radiosensitizing metabolite, enhances PD-1 inhibitor efficacy by inducing immunogenic cell death and activating STING-dependent antitumor immunity (119, 125). Additionally, plant-derived nanoparticles (e.g., ginger exosome-like nanoparticles) increase DHA via Lachnospiraceae modulation, which binds the PD-L1 promoter to suppress its transcription and overcome resistance (125).

**Figure 3:**
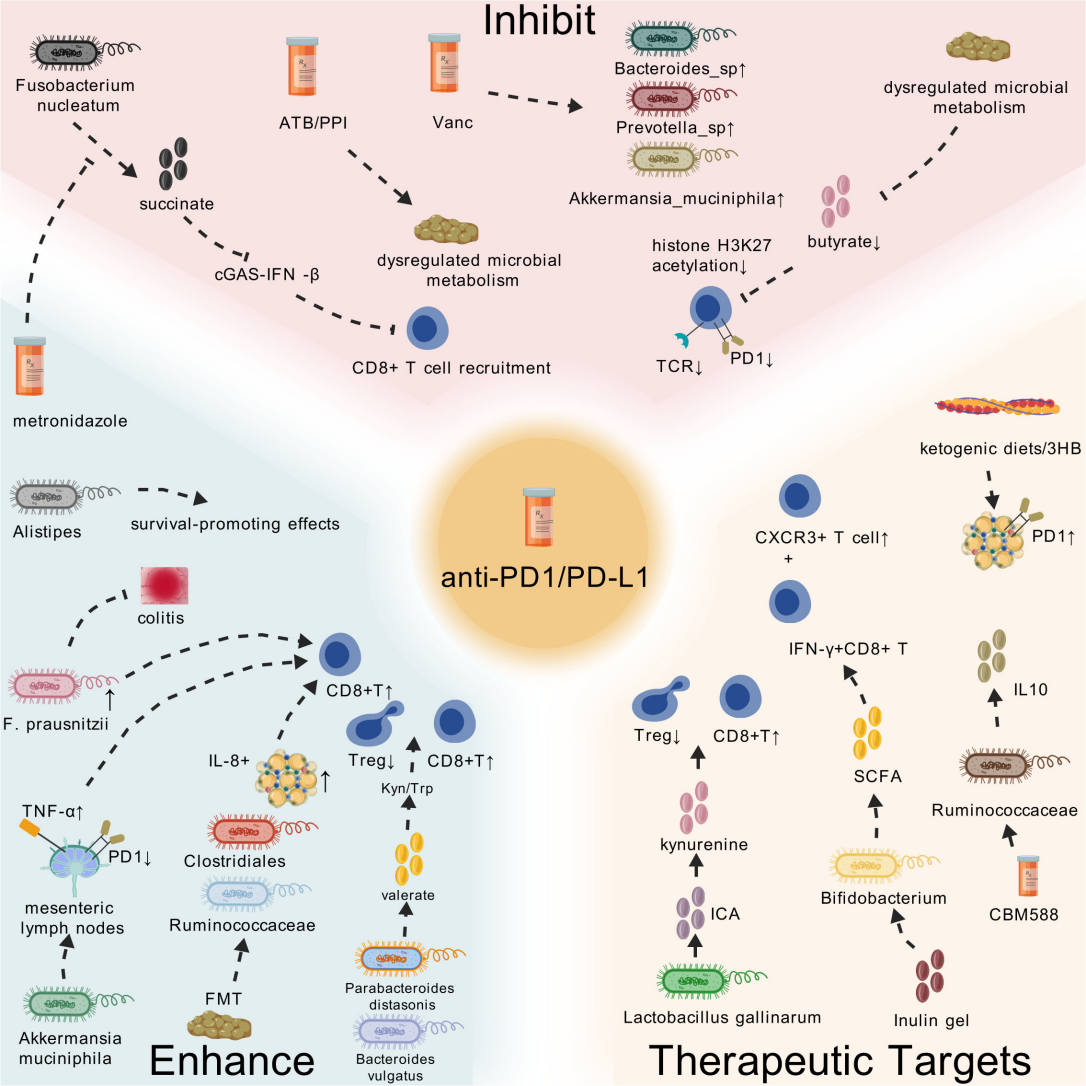
Microbiota and PD1/PD-L1 immunotherapy. In tumors, dysregulated microbial metabolism or drug-induced dysregulated microbial metabolism can inhibit the efficacy of PD1/PD-L1 monoclonal antibodies through a cascade reaction. Some probiotics such as Akkermansia Muciniphila, Bacteroides vulgatus, Ruminococcaceae, etc. can promote or activate the anti-tumor immune response through a cascade reaction. Taking probiotics, FMT, KD, etc. can affect the anti-tumor immune response by regulating the microbial composition.

The gut microbiota profoundly influences PD1/PD-L1 immunotherapy outcomes through taxon-specific interactions, metabolite signaling, and immune pathway modulation. Enriching beneficial taxa (e.g., Akkermansia, Lactobacillus gallinarum) and depleting resistance-associated species (e.g., Fusobacterium nucleatum) via dietary, pharmacological, or microbial interventions holds transformative potential for personalized cancer immunotherapy (**Figure 3**). We summarize more relevant studies in **Table 1**.

**Table 1:** Microbiota and PD1/PD-L1 Immunotherapy.

Cancer type	Microbiota/metabolites	Mechanism and function	Ref
Lung cancer	valeric acid/L-kynurenine	Ginseng polysaccharides (GPs) increase the level of valeric acid, a microbial metabolite, and reduce the immunosuppressation-related metabolites L-kynourine (KYN) and the Kyn/Trp ratio, thereby inhibiting Tregs, activating effector T cells, and enhancing the immune response. GPs combined with PD-1 monoclonal antibody is a promising sensitization strategy for immunotherapy, and the gut microbiota is expected to serve as a novel biomarker for predicting therapeutic responses.	(117)
Melanoma	FMT derived from reacters	FMT from responders to immunotherapy has caused rapid and continuous changes in the intestinal microbiota, increased the abundance of microbiota related to the immune response, enhanced the activation of CD8+ T cells, reduced MDSCs expressing IL-8, and further improved the TME. FMT combined with PD-1 inhibition is a promising anti-drug resistance immunotherapy strategy.	(56)
Colorectal cancer	Bacteroides acidificus	Gegen Qinlian Decoction combined with PD-1 inhibitors boosts immunotherapy for MSS CRC. The treatment enriches gut bacteria like Bacteroides acidificus and alters lipid metabolism (glycerophospholipids/sphingolipids). It also increases CD8+ T cells, enhances IFN-γ/IL-2 expression, and reduces PD-1 levels, reactivating T cells. This microbiome-metabolism-immune approach offers a new strategy for MSS CRC.	(133)
Colorectal cancer	Lactobacillus gallinarum	The probiotic Lactobacillus gallinarum can significantly enhance the therapeutic effect of CRC on PD-1 inhibitors by regulating the immune microenvironment. Mechanistically, the key metabolite ICA produced by Lactobacillus gallum can inhibit the expression of IDO1 and reduce the generation of the immunosuppressive metabolite Kyn. ICA also antagonizes KYN-induced differentiation of CD4+ Tregs by competitively binding to AHR, thereby reducing Treg infiltration and enhancing the effector function of CD8+ T cells. In CRC models with high MSI and low MSI, both Lactobacillus galactica and ICA effectively improved the efficacy of anti-PD-1, while exogenous Kyn could reverse this enhancing effect.	(123)
Colorectal cancer	Fusobacterium nucleatum	F. Succinic acid derived from nucleatum can inhibit the CGAS-interferon -β pathway, hindering the migration of CD8+ T cells to the TME, thereby weakening the anti-tumor immune response. Transplanting fecal microbiota from responders with low Clostridium nucleatum levels into mice can enhance their sensitivity to PD-1 antibody treatment. However, the high level of Clostridium nucleatum does not. After using metronidazole to reduce the abundance of F. nucleatum in the intestine, the succinic acid level can be reduced and the sensitivity to immunotherapy can be restored. The study revealed the key role of Clostridium nucleatum - succinic acid - immune pathway in immune resistance of CRC and provided potential intervention strategies.	(119)
Colorectal cancer	Akkermansia muciniphila	This study found that the intestinal symbiotic bacteria Akkermansia muciniphila and its outer membrane protein Amuc_1100 have immunomodulatory and anti-tumor effects in colitis and colitis-associated CRC. Mechanologically, A. muciniphila or Amuc_1100 can reduce the infiltration of pro-inflammatory macrophages and CTLs in the intestine and immune organs, thereby alleviating colitis. Meanwhile, in CAC, it promotes the expansion and activation of CTLS and enhances anti-tumor immunity. Especially in mesenteric lymph nodes, they inhibit tumorigenesis by increasing the expression of TNF-α and down-regulating PD-1 and enhancing CTL function.	(118)
Gastrointestinal cancer	Prevotella/Bacteroides	Patients with an elevated ratio of Prevotella to Bacteroides are more likely to respond to immunotherapy, and the abundances of Prevotella, Ruminococcaceae and Lachnospiraceae in the responders increase significantly. Immune responders are rich in metabolic pathways related to nucleotide synthesis, lipid metabolism, glucose metabolism and SCFAs fermentation. In particular, the bacterial communities capable of generating SCFA, such as Eubacterium, Lactobacillus and Streptococcus, are positively correlated with the efficacy of anti-PD-1 /PD-L1 treatment.	(134)
/	/	Microbial abundance based on strains is more effective in predicting the treatment response and progression-free survival of ICB than traditional species-level microbial abundance or clinical factor models. The characteristics of the strains are universal among different cancer types and countries, provided that the ICB treatment regimens are consistent. This indicates that future diagnostic and therapeutic strategies related to the gut microbiome should be personalized based on the ICB treatment plan rather than the type of cancer.	(135)
Malignant mesothelioma	Intestinal microbiota	Some genera of the intestinal microbiota are positively correlated with the therapeutic response, especially with CD8+ T cell infiltration, but negatively correlated with the intrinsic drug resistance factors of the tumor (such as UPD, HRD and CD68+ monocyte infiltration).	(136)
Biliary tract cancer	Alistipes, Bacilli, Lactobacillales and Pyrrolidine	The intestinal microbiota and metabolites affect the immunotherapy response of patients with biliary tract carcinoma, and there are significant differences in bacteria and metabolites between the long-term clinical benefit group and the non-long-term clinical benefit group. Alistipes is positively correlated with survival, while Bacilli, Lactobacillales and Pyrrolidine are negatively correlated with survival.	(121)
Colorectal cancer	Faecalibacterium prausnitzii	The abundance of F. prausnitzii in the intestines of colitis patients treated with ICI is relatively low. In mouse experiments, the dual blockade of ICI (CTLA4 and PD-1) exacerbated colitis and triggered more infiltration of immune cells. F. prausnitzii can alleviate colitis caused by ICI, enhance the tumor immune response at the same time, and promote the anti-tumor effect. Furthermore, F. prausnitzii improves the overall effect of ICI treatment by increasing the diversity of intestinal microbiota, regulating the microbial composition, and inhibiting the growth of potential pathogens. In conclusion, F. prausnitzii not only alleviated ICI-induced colitis but also enhanced the anti-tumor activity of immunotherapy.	(120)
Rectal cancer	Methylglyoxal	MG is a metabolite related to the intestinal microbiota and can effectively predict the response of patients with locally advanced rectal cancer to preoperative radiotherapy. MG enhances the efficacy of radiotherapy by stimulating intracellular reactive oxygen species, reducing tumor hypoxia, increasing DNA double-strand breaks, and activating the interferon gene pathway. Meanwhile, MG promotes immunogenic cell death caused by endoplasmic reticulum stress and increases the infiltration of CD8+ T cells and natural killer cells in the TIM. Ultimately, MG combined with anti-PD1 therapy can produce a lasting and complete response at the tumor site undergoing radiotherapy and the unirradiated site.	(125)
Prostate cancer	SCFA	The combined treatment of icariin and curcumin (CUR) can significantly reduce the volume and weight of tumors in mice and change the composition of the intestinal flora. The treatment by influencing the SCFAs and DNMT1 IGFBP2 / EGFR/STAT3 / PD - L1 pathway, enhance the T cell immune reaction, increase the CD3 + CD8 + IFN - gamma, CD3 + CD8 + cells Ki67 positive rate, Meanwhile, the levels of IFN-γ and IFN-α in the serum were increased. The research also found that SCFAs promote tumor development and increase the expression of IGFBP2, while DNMT1/IGFBP2 promotes cell migration and proliferation. icariin-CUR inhibits the progression of PCa by suppressing the expression of DNMT1/IGFBP2. Overall, icariin-CUR provides potential therapeutic strategies for prostate cancer by regulating immune responses, metabolism and gut microbiota.	(137)
Breast cancer	Intestinal flora	Flaxseed lignan (FL) is converted into enterolactone through the intestinal microbiota, which can down-regulate the CD38 gene and inhibit the malignant behavior of BC cells. CD38 is a key gene related to immunosuppression and resistance to PD-1/PD-L1 blockade. FL treatment increased the number of CD3+, CD4+ and CD8+ cells in the TIM, while reducing F4/80+ cells. FL combined with PDi (FLcPDi) further regulates TIM and significantly increases the abundance of Akkermansia in the gut microbiota. Importantly, the increase of Akkermansia enhanced the response of mice treated with antibiotics to PDi. Studies have shown that FL not only inhibits the progression of BC but also enhances the anti-cancer effect of PDi by regulating the intestinal microbiota and the host immune system.	(127)
/	Intestinal microbiota/SCFAs	Inulin, as a common dietary fiber, can effectively regulate the intestinal microbiota as an oral gel and enhance the anti-tumor effect of ICIs (such as α-PD-1). Oral inulin gel therapy can increase the abundance of key symbiotic microorganisms and their SCFA metabolites in the intestine, and enhance the systemic response of memory T cells. Specifically, the treatment increased the recall response of interferon -γ+CD8+ T cells and promoted the formation of stem cell-like T cytokine -1+PD-1+CD8+ T cells in the TME.	(124)
Non-small-cell lung carcinomas	Ruminococcal bacteria	cbm588 can improve intestinal immune homeostasis by altering the intestinal flora, especially by increasing the abundance of ruminococcal bacteria, and effectively enhance the response to PD-1 blockade. cbm588 treatment promoted the expression of indoleamine 2, 3-dioxygenase 1 and IL-10 in the colon and enhanced the ability of lamina propria monocytes in tumor carriers to secrete IL-10. However, blocking the IL-10 signaling pathway enhances the ability of CD8+ T cells to secrete interferon -γ.	(126)
Gastric cancer	Lactobacillus	The changes of the intestinal microbiome are closely related to gastric cancer and its precancerous lesions. The abundance of Lactobacillus is positively correlated with the reactivity and PFS of PD-1/PD-L1 immunotherapy.	(138)
Breast cancer	Bifidobacterium, Faecalibaculum and Lactobacillus	Fucoidan combined with anti-PD-1 treatment significantly enhanced the anti-tumor effect and improved the composition of the intestinal microbiota by increasing beneficial bacteria (such as Bifidobacterium, Faecalibaculum and Lactobacillus). After antibiotic intervention in the intestinal flora, the anti-tumor effect of fucoidan was significantly reduced. Fucoidan reverses metabolic disorders in breast cancer models through tryptophan metabolism and glycerophospholipid metabolism pathways, significantly increasing the content of SCFAs (particularly acetic acid and butyric acid), which improves the function of effector T cells and inhibits the generation of Treg cells.	(139)
/	Christensen bacteria	Specific strains of Christensen bacteria can selectively enhance the anti-cancer effect of off-target low-dose intestinal irradiation and PD-L1 blockade, and promote the migration of intestinal dendritic cells expressing PD-L1 to tumor-draining lymph nodes.	(140)
Non-small cell lung cancer	Faecalibacterium/ SCFA	The improvement of intestinal microbiota diversity is significantly associated with a positive response to ICIs treatment. The number of Faecalibacterium in the intestinal flora of the respondents increased significantly, and at the same time, the levels of SCFAs, especially butyric acid, acetic acid and caproic acid, also increased. FMT can effectively delay tumor progression and enhance the effect of immunotherapy, although it does not change the expression of PD-L1 in mouse tumor tissues. These results indicate that the diversity of the intestinal microbiota and the level of SCFA are closely related to the effect of immunotherapy, and FMT can be used as a potential strategy to enhance the effect of immunotherapy.	(141)
Oral squamous cell carcinoma	Porphyromonas, Prevotella and Clostridium subcluster XIVa	Porphyromonas and Prevotella significantly increased in patients with OSCC, while in the PD-L1 positive group, the abundance of Clostridium subcluster XIVa in the intestinal flora was significantly higher than that in the PD-L1 negative group. Studies also show that there is an ecological imbalance in the oral and intestinal microbiota of OSCC patients. The results suggest that probiotics and synthetic bacteria targeting these disease-specific microbiota may offer possibilities for new cancer treatment methods.	(142)
/	Collinsella aerofaciens	Collinsella aerofaciens is usually associated with pro-inflammatory responses and adverse health outcomes, but there are significant exceptions to its favorable response to PD-1/PD-L1 cancer immunotherapy. The glycosylated glyceride compound CaLGL-1 produced by Collinsella aerofaciens has a β -galactofuran sugar head group with acetal and can activate the immune response through the TLR2-dependent signaling pathway. Furthermore, this compound is transformed from plasmalogen, which is synthesized by bacteria, in a low pH environment.	(143)
/	Intestinal microbiota	There were significant differences in the composition and diversity of the microbiota between the response group (R) and the non-response group (NR) receiving immunotherapy. In Group R, genera such as Paraphylbacillus, Clostridium and Bifidobacterium dentatum were enriched, while in group NR, genera such as Bacteroides multiflorum and Nocardia were relatively abundant. The abundance of Weissella in group R increased significantly at 6 weeks, while the abundance of Fusobacterium and Anaerotruncus in group NR increased significantly at 12 weeks. Linear discriminant analysis showed that there were also significant differences in the intestinal microbiota between the immune-related adverse events (AE) group and the non-AE group. The AE group was mainly enriched with hard-walled microbiota.	(144)
/	Prevotella and Faecalis	Gut microbiota α-diversity shows positive correlation with prolonged overall survival (OS) in cancer patients receiving ICI therapy. The microbiome composition of responders resembles that of healthy individuals, with specific bacteria like Prevotella and Faecalibacterium being associated with better treatment outcomes.	(145)
Triple-negative breast cancer	Intestinal microbiota	Higher Faith's phylogenetic diversity (PD)-based α-diversity was significantly associated with prolonged PFS, particularly in patients receiving atezolizumab chemotherapy, while no such association was observed in the placebo group. Furthermore, patients with high Faith PD levels showed lower PFS risk, whereas no significant difference was found in the low Faith PD group.	(146)
Non-small cell lung cancer	Odor Bacillus, Gordon Bacillus and Clostridium strictorium	Responders exhibited decreased abundance of Odoribacter and Gordonibacter before and after treatment, along with increased abundance of Clostridium sensu stricto. In contrast, non-responders showed elevated abundance of Prevotella and reduced abundance of Akkermansia after treatment. The study suggests that dynamic changes in gut microbiota may serve as a non-invasive biomarker to predict response to PD-1/PD-L1 blockade therapy in NSCLC, providing a theoretical basis for larger prospective studies.	(147)
Lung cancer	Intestinal flora and metabolites	The ICI-benefited group showed increased levels of propionate and butyrate/isobutyrate. Specifically, probiotic species mediated butyrate production via the acetyl-CoA pathway. Compared to patients with immune-related adverse events (irAEs), responders exhibited a higher abundance of microbial epitopes associated with autoimmune diseases and lung cancer antigens. The benefited group had elevated levels of acetate, propionate, and butyrate, which correlated with prolonged PFS and lower tumor progression risk. Long-term ICI responders harbored gut microbiota with high butyrate production, and molecular mimicry of tumor and autoimmune antigens by the microbiota may have contributed to the therapeutic effect.	(122)
Small cell lung cancer	Intestinal microbiota	Responding patients (R group) after immunotherapy showed significantly higher gut microbiota α-diversity than non-responding patients (NR group), with distinct microbial composition between the two groups. The R group was enriched with Faecalibacterium, Clostridium_sensu_stricto_1, and Ruminococcus_torques, while the NR group showed enrichment of Dubosiella and coriobacteriaceae_UCG-002. Additionally, R group patients exhibited upregulated SCFAs post-treatment, and metabolites associated with PD-L1 expression and PD-1 checkpoint pathway in cancer were enriched in KEGG pathways.	(148)
Lung cancer	Intestinal microbiota	Metformin enhances the anti-tumor effect of PD-L1 antibodies, and this effect depends on the presence of gut microbiota. Metformin improves anti-tumor immune responses and enhances intestinal mucosal integrity by modulating the gut microbiota. After antibiotic depletion of gut microbiota, the combined therapeutic effect of metformin and PD-L1 antibodies disappears, indicating the crucial role of gut microbiota in their anti-tumor action.	(149)
Colorectal cancer	Clostridium butyricum/Akkermansia muciniphila	mbined treatment with Clostridium butyricum and Akkermansia muciniphilasignificantly reduced inflammatory infiltration of macrophages and cytotoxic T lymphocytes, modulated anti-tumor immune responses, and suppressed CRC development. Additionally, it enhanced the sensitivity of mice to ICIs (anti-PD-L1), improved the anti-tumor efficacy of immunotherapy, and markedly prolonged survival in mice.	(150)
/	Lachnospiraceae and Ruminococcacea	With the development of acquired resistance, certain microbiota associated with the efficacy of ICIs (e.g., Lachnospiraceae, Ruminococcaceae) were observed to decline. Additionally, butyrate-producing bacteria were significantly reduced in the acquired resistance group. In contrast, the non-acquired resistance group showed a transient decrease in the abundance of IgA-coated bacteria during the initial and sustained treatment phases.	(151)
/	Butyric acid	The combined use of polygalacturonic acid (PGA)​and lipopolysaccharide-coated PLGA-PEG-PLGA (LPS/PPP)​​ significantly increased the abundance of ​SCFAs (e.g., butyrate)​​ in the gut and enhanced immune responses. When ​PGA_LPS​ was administered in combination with ​anti-PD-L1 antibody (αPD-L1)​, it effectively suppressed the growth of ​CT26 and 4T1 tumors, induced ​T-cell responses, and inhibited ​T-cell exhaustion.	(93)
/	Clostridium and Lactobacillus johnsonii	Bilberry anthocyanins promoted the proliferation of beneficial gut bacteria such as Clostridium and Lactobacillus johnsonii, enhanced gut microbiota diversity, and consequently improved the immune microenvironment while boosting response to immunotherapy.​	(152)
Bladder cancer	Blautia	The gut probiotic Blautia (Blautia coccoides) significantly enhances the anti-tumor efficacy of PD-1 ICIs in bladder cancer treatment. Mechanistically, Blautia produces the bioactive metabolite trigonelline, which suppresses the Wnt/β-catenin signaling pathway. This promotes CD8^+^ T cell infiltration and enhances their anti-cancer activity within the TME, leading to potent tumor growth inhibition. These findings position Blautia and its metabolites as promising adjuvant therapeutics to boost the clinical effectiveness of PD-1 inhibitors.​	(153)
Colorectal cancer	Prevotellaceae	Patients with higher abundance of Prevotellaceae exhibit enhanced immune cell infiltration and more robust anti-tumor responses. Mechanistically,​​ Prevotellaceae ​may potentiate the efficacy of anti-PD-L1 therapy by modulating immune factors​ (e.g., IFN-γ, IL-2) ​in the TME, thereby amplifying antitumor immunity.​	(154)
Colorectal cancer	Limosilactobacillus, Desulfovibrio	The herbal preparation Chang-Wei-Qing combined with PD-1 inhibitors regulates the gut microbiota by enhancing microbial diversity, enriching beneficial bacteria such as Limosilactobacillus and Bifidobacterium, and reducing pathogenic bacteria like Desulfovibrio, thereby improving gut dysbiosis. Simultaneously, this treatment downregulates pro-inflammatory metabolites associated with the NF-κB signaling pathway, enhances intestinal barrier function (including tight junctions and cellular integrity), upregulates protective genes, and suppresses detrimental factors related to innate immune responses.	(155)
/	Intestinal microbiota	The use of antibiotics (ATB) and/or proton pump inhibitors (PPI) prior to ICIs treatment significantly impacts therapeutic outcomes and survival prognosis in cancer patients. ATB and PPI disrupt the gut microbiota, thereby impairing the immune system's response to ICI therapy. Patients receiving ATB and/or PPI exhibit significantly reduced PFS and OS, with the highest PFS risk observed in those concurrently using both medications.	(156)
Colorectal cancer	Bacteroides_sp, Prevotella_sp and Akkermansia_muciniphila	Antibiotic treatment can attenuate the tumor growth-inhibitory effects of PD-1 antibodies, with specific bacterial groups such as Bacteroides spp., Prevotella spp., and Akkermansia muciniphila being enriched in different antibiotic groups. Notably, in the vancomycin group, metabolites were found to be enriched in the glycerophospholipid metabolism pathway. Further analysis revealed that gut microbiota alterations modulate the expression of immune cytokines (e.g., IFN-γ and IL-2) in the TME by influencing glycerophospholipid metabolism, thereby regulating the therapeutic efficacy of PD-1 antibodies.	(157)
Urothelial carcinoma	Intestinal microbiota	Antibiotic use was significantly associated with worse OS and PFS in patients treated with atezolizumab. However, no impact of antibiotics on survival outcomes was observed in the chemotherapy group.	(158)
Lung adenocarcinoma	Intestinal microbiota	The beneficial effects of sintilimab are associated with changes in gut microbiota diversity, suggesting that intestinal microbes may play a significant role in its immunomodulatory mechanism. The combination of sintilimab with prebiotics may inhibit lung adenocarcinoma and improve the safety profile of Sin through modulation of the gut microbiota.	(159)

### 4.6 Bridging the chasm between preclinical research and clinical application​

Despite a wealth of convincing preclinical studies elucidating the mechanisms by which the microbiota regulates the PD-1/PD-L1 axis, its translation into clinical application remains challenging, often yielding inconsistent results (160, 161). This “translational chasm” stems from multiple factors. First, there are fundamental differences in research models. Preclinical studies predominantly use inbred mice reared under germ-free or standardized conditions, which have low-diversity and well-defined microbiomes (160). In contrast, the human microbiome is profoundly shaped by genetics, diet, antibiotic history, and environment, resulting in exceptionally high inter-individual heterogeneity (162, 163). Furthermore, mouse tumor models are often homogeneous and progress rapidly, whereas human tumors undergo long-term evolution, exhibiting high heterogeneity and a complex immune-edited microenvironment (160, 161). Consequently, single bacterial strains or simple consortia that show significant efficacy in mouse models often struggle to colonize or exert their intended effects within the complex ecosystem of humans (163). Second, the vast inter-individual heterogeneity poses a major obstacle to clinical translation. Factors such as the patient’s baseline microbiome composition, immune status, genetic background, and concomitant medications collectively lead to highly variable responses to microbial interventions (162). Research indicates that the colonization capacity of even the same probiotic strain depends on an individual’s inherent gut microbiota structure and physiological conditions (e.g., intestinal transit time) (163). This distinction between “permissive” and “non-permissive” hosts explains why clinical trial results are often inconsistent and underscores the necessity for personalized, precision interventions in the future. Finally, limitations in study design and methodology cannot be overlooked. Many preclinical studies have small sample sizes, failing to mimic the scale and population diversity of clinical trials. In microbiome analysis, the lack of standardized protocols for sample processing, sequencing, and data analysis makes direct comparison of results across studies difficult. Current 16S rRNA sequencing based on relative abundance cannot distinguish bacterial viability or absolute quantity, potentially failing to accurately reflect changes in the functional microbial community (163). To overcome these challenges, future research should focus on: 1) Developing animal models (e.g., humanized models) that more closely recapitulate human disease states; 2) Conducting large-scale, multi-center prospective clinical studies integrated with multi-omics technologies to deeply characterize causal functional bacterial strains and their metabolites; 3) Establishing standardized protocols for microbiome analysis and management; 4) Advancing precision stratification of patients based on their baseline microbial features to enable personalized interventions. Only through in-depth interdisciplinary collaboration can we successfully bridge this translational chasm and realize genuine microbiome-based precision immunotherapy.

### 4.7 Clinical translation progress and challenges ​

Breakthrough discoveries from preclinical research are being rapidly translated into real-world therapeutic strategies through a series of clinical trials, aiming to validate the feasibility of enhancing the efficacy of PD-1/PD-L1 inhibitors by modulating the microbiota. Among these strategies, fecal microbiota transplantation (FMT) is the most extensively studied intervention. In patients with advanced melanoma, several early-phase clinical trials have demonstrated its potential. For PD-1 inhibitor-refractory patients who did not respond initially, infusion of fecal microbiota from responders (e.g., in trial NCT03341143) could reshape the gut microbiota and reverse the immunosuppressive tumor microenvironment in a subset of patients (approximately 40%), thereby overcoming drug resistance (56). More prospective explorations have combined FMT from healthy donors with PD-1 inhibitors as first-line therapy (NCT03772899), showing that this regimen has a favorable safety profile and achieved an objective response rate as high as 65%, suggesting FMT’s potential to prevent or delay the onset of resistance (164). Furthermore, small-scale studies in patients with anti-PD-1 refractory metastatic melanoma also confirmed the safety of FMT combination therapy and observed favorable changes in immune cell infiltration within the tumor microenvironment (165).

Beyond directly transplanting the entire microbial community, more precise microbial modulation strategies are under development. For instance, a multicenter, randomized, placebo-controlled Phase I trial (NCT03817125) evaluated the combination of SER-401, a spore-based formulation rich in Firmicutes, with nivolumab. Although this study was underpowered due to enrollment challenges during the pandemic, its translational analysis provided key insights: an improperly designed antibiotic pretreatment regimen intended to empty ecological niches might disrupt the microbiota and impair immunity, consequently weakening the subsequent efficacy of immunotherapy (166). Addressing the clinical challenge of antibiotic-induced dysbiosis, an innovative study tested the colon-targeted adsorbent DAV132 (NCT03678493). This device effectively reduced fecal antibiotic levels without affecting plasma antibiotic concentrations, thereby protecting gut microbiota diversity in healthy volunteers. Preclinical models further confirmed that fecal transplants from DAV132-protected volunteers could prevent antibiotic-associated resistance to anti-PD-1 therapy (167).

Notably, the influence of the microbiota may extend beyond traditionally “hot” tumors. In the ALICE trial for triple-negative breast cancer, higher baseline gut microbiota alpha diversity (Faith’s PD) was significantly associated with improved efficacy of atezolizumab (anti-PD-L1) combined with chemotherapy, suggesting that microbiome features could serve as predictive biomarkers, even in PD-L1-negative patients (146).

However, translating these preliminary successes into universal clinical protocols faces significant challenges. Existing trials are mostly early-phase, single-arm studies with small sample sizes and heterogeneous outcomes. Donor screening for FMT, standardization of preparations, long-term safety, and the substantial inter-individual heterogeneity of microbiota are pressing issues that need resolution (166, 168–171). Future directions require larger-scale randomized controlled trials, biomarker-based precise patient stratification, and the development of standardized synthetic microbial consortia composed of well-defined functional strains, ultimately enabling the precise application of microbiota modulation in cancer immunotherapy.

## 5 Clinical implications and future directions

The growing body of evidence indicating that the gut microbiota can modulate immune responses has led to a novel perspective in cancer immunotherapy. Specifically, the gut microbiota’s ability to influence the PD1/PD-L1 immune checkpoint pathway offers an exciting avenue for enhancing the efficacy of existing therapies. Various studies have shown that specific microbial species, including Bifidobacterium, Akkermansia muciniphila, and Faecalibacterium prausnitzii, can stimulate the immune system and increase the response to ICIs (89, 102, 120). By modulating the composition of the microbiota, either through dietary interventions, probiotics, or FMT, it may be possible to enhance the anti-tumor immune response and improve the effectiveness of ICIs (164, 172, 173). The manipulation of the microbiota could work in several ways to boost immune therapy efficacy. First, beneficial microbes can activate immune cells such as dendritic cells, macrophages, and T cells, promoting a more robust immune response (174, 175). Second, the metabolites produced by these microbes, such as SCFAs, can influence immune cell function and promote immune tolerance, potentially counteracting the immune suppression induced by tumors. Third, modulating the microbiota could lead to changes in the TME by either enhancing immune cell infiltration or modifying the immune checkpoint signaling landscape, ultimately improving tumor recognition and elimination (164, 172, 173). This approach also holds the potential to overcome some of the challenges associated with ICIs, such as primary resistance or relapse after initial success. As such, integrating microbiota modulation into cancer immunotherapy could represent a promising strategy to increase the clinical success of these therapies.

As cancer therapies become increasingly personalized, the integration of microbiome modulation into precision medicine offers new opportunities for tailoring treatments to individual patients. The composition of the microbiota can vary widely between patients, and this variability has been shown to influence the response to immunotherapy. By analyzing a patient’s microbiome profile, clinicians could predict the likely success of ICIs and personalize the treatment strategy. In the context of precision cancer immunotherapy, microbiome modulation could be used to optimize treatment outcomes. For instance, specific microbial populations that are known to promote anti-tumor immunity could be administered through probiotics or FMT to enhance the patient’s immune response. Additionally, the microbiome could help predict which patients are more likely to respond to therapies targeting the PD1/PD-L1 axis, facilitating a more targeted approach to immunotherapy and reducing unnecessary side effects for non-responders. By combining microbiome profiling with other factors such as genetic and environmental data, precision medicine could enable more effective and individualized cancer treatments (176–178). This could lead to the development of microbiota-based biomarkers that help identify the most suitable treatment for each patient, thereby improving clinical outcomes and minimizing trial-and-error approaches.

Despite the promising potential of microbiome modulation in cancer immunotherapy, several challenges remain in translating these findings into clinical practice. One major challenge is the lack of standardized protocols for microbiome-based interventions, such as the use of probiotics, prebiotics, or FMT. The composition and function of the microbiota can be highly individual, and what works for one patient may not be effective for another. Additionally, the long-term safety and stability of microbiome-based treatments need to be thoroughly evaluated to ensure that they do not lead to adverse effects, such as the development of dysbiosis or other immune-related complications. Another challenge is the complexity of the microbiota’s interactions with the host immune system. While research has identified key microbes and metabolites that influence immune responses, the precise mechanisms by which microbiota modulate the immune system remain incompletely understood. Future studies should focus on identifying specific microbial species or combinations that have consistent and reproducible effects on immune checkpoint signaling and tumor immunity. Furthermore, more research is needed to determine the optimal strategies for microbiota modulation, including the appropriate dosage, timing, and duration of interventions. Finally, clinical trials are necessary to assess the safety and efficacy of microbiome-based interventions in combination with ICIs. These trials will need to include diverse patient populations to determine whether the effects of microbiome modulation are consistent across different cancer types, stages, and treatment regimens. Additionally, the development of microbiome biomarkers will be crucial for predicting which patients are most likely to benefit from microbiome-based therapies, paving the way for personalized immunotherapy approaches.

Despite compelling mechanistic evidence from preclinical studies regarding microbiota-mediated regulation of the PD-1/PD-L1 axis, translating these findings into clinical practice faces significant challenges. A major obstacle lies in the fundamental differences between preclinical models and the human context. Firstly, most mechanistic studies rely on inbred mice housed under germ-free or standardized conditions, whose microbiome diversity is far lower than the highly heterogeneous human microbiome shaped by genetics, diet, lifestyle, and antibiotic history. Secondly, commonly used mouse tumor models (e.g., transplanted tumor models) typically progress rapidly and possess a relatively simplistic immune context within the tumor microenvironment. In contrast, human cancers evolve over years, exhibiting extreme heterogeneity and immune editing, which fosters a more complex immunosuppressive milieu. These discrepancies result in many microbiota-based interventions (such as specific probiotics or microbiota transplantation), which show significant effects in mouse models, demonstrating only modest or inconsistent efficacy in human clinical trials (179, 180). Furthermore, correlations observed in human studies between microbial signatures and treatment response do not equate to causality. Although FMT)experiments provide some causal evidence, definitive proof requires more targeted interventions (e.g., defined microbial consortia) and a clear elucidation of their downstream molecular mechanisms. Therefore, when interpreting available data, the translational potential of preclinical findings must be assessed critically, and more resources should be dedicated to validating the relevance of these mechanisms in human biology.

The core challenge in successfully translating microbial-based regulatory strategies into clinical practice lies in managing their profound context-dependency. A significant limitation of current research is the low degree of overlap observed in “beneficial” microbial signatures associated with immunotherapy response across different patient populations and cancer types (181). This inconsistency likely stems from substantial variations in geography, diet, genetic makeup, and baseline microbiome composition among populations. For instance, while *Akkermansia muciniphila* is considered a strain that enhances the efficacy of PD-1 blockade in lung cancer and melanoma, its role in the context of inflammatory bowel disease is more complex. The key for future research lies in shifting the focus from searching for universal “one-size-fits-all” bacterial strains toward defining functional microbial niches within specific host backgrounds and particular tumor types. This requires employing multi-omics approaches that integrate metagenomics, metabolomics, and host immune profiling to map the precise “microbe-immune-tumor” interaction network, ultimately enabling truly personalized microbial interventions.

Before advancing toward clinical application, the field must address several core limitations. The primary issue is the predominance of correlation over causation. Although numerous studies have linked specific microbial signatures to immunotherapy efficacy, causal evidence—aside from FMT—directly demonstrating that particular bacteria are drivers of treatment response remains limited. Many so-called “beneficial bacteria” may be markers of a healthier immune system rather than active participants. Secondly, there is a fallacy of reductionism. Current research often focuses on isolating single “star” bacterial strains or metabolites, but microbiota function as complex ecological networks. Strategies targeting a single component may disrupt the overall balance of the community, and their effects could be negated by compensatory mechanisms. Finally, a major obstacle is the lack of technical standardization. The absence of uniform protocols—from sample collection and DNA sequencing to bioinformatic analysis—makes direct comparison of results across studies difficult and contributes to the irreproducibility of many findings.

Future research should strive to overcome these limitations and shift from describing associations to dissecting underlying mechanisms. The priorities are as follows: First, elucidate causal mechanisms by utilizing tools such as genetically engineered bacteria, conditional knockout animal models, and advanced co-culture systems like organoids to precisely trace the complete signaling cascade from microbial signals to host immune pathways (e.g., PD-1/PD-L1). Second, actively embrace complexity by moving from studying single strains toward defining “minimal functional communities,” and employ systems biology and artificial intelligence approaches to understand intra-community interactions and their collective impact on the host. Finally, promote clinical translation. Future clinical trials should incorporate standardized microbiome analysis coupled with immune monitoring to identify predictive biomarkers. The focus should be on developing mechanism-based, tailored interventions—such as next-generation probiotics or targeted metabolite therapies designed for the microbiome characteristics of non-responders—rather than simplistic “one-size-fits-all” supplementation.

## 6 Conclusion

Microbiota-based approaches represent an exciting frontier in cancer immunotherapy. By unraveling the intricate relationships between the microbiota and immune checkpoint pathways, we can develop more effective, personalized treatments that improve patient outcomes and help overcome the limitations of current immunotherapy regimens. The future of cancer immunotherapy lies in harnessing the power of the microbiota to enhance tumor immunity and combat cancer resistance.
